# A Novel UHPLC–MS/MS Method for the Measurement of 25-Hydroxyvitamin D3 in Canine Serum and Its Application to Healthy Dogs

**DOI:** 10.3390/ani14010062

**Published:** 2023-12-23

**Authors:** Anisa Bardhi, Carla Giuditta Vecchiato, Maria Chiara Sabetti, Antonio Maria Tardo, Kateryna Vasylyeva, Giacomo Biagi, Marco Pietra, Andrea Barbarossa

**Affiliations:** 1 Department of Veterinary Medical Sciences, University of Bologna, 40064 Ozzano dell’Emilia, Italy; anisa.bardhi@unibo.it (A.B.); antoniomaria.tardo2@unibo.it (A.M.T.); kateryna.vasylyeva2@unibo.it (K.V.); giacomo.biagi@unibo.it (G.B.); marco.pietra@unibo.it (M.P.); andrea.barbarossa@unibo.it (A.B.); 2 Department of Veterinary Sciences, University of Parma, 43124 Parma, Italy; mariachiara.sabetti@unipr.it; 3 Health Sciences and Technologies-Interdepartmental Centre for Industrial Research (CIRI-SDV), University of Bologna, 40064 Ozzano dell’Emilia, Italy

**Keywords:** assay, calcitriol, calcidiol, dogs, reference interval

## Abstract

**Simple Summary:**

Vitamin D deficiency and toxicity are common and well-recognized conditions in dogs. The metabolite 25-hydroxyvitamin D3 is the main endogenous biomarker for the evaluation of vitamin D3 status. In this study, we developed a simple analytical method for 25-hydroxyvitamin D3 quantification in canine serum. The method was validated following the current European guidelines and yielded satisfactory results, representing a new tool for supporting clinicians in the correct diagnosis and monitoring of vitamin D3 status in dogs.

**Abstract:**

Several studies have shown the importance of vitamin D3 supplementation in small animals. In dogs, a low vitamin D3 status is associated not only with bone metabolism but also with different kinds of disorders, such as congestive heart failure, gastrointestinal diseases, chronic kidney diseases, and some types of cancer. However, it is crucial to maintain balance and monitor the introduction of this essential nutrient through the diet because over-supplementation can result in toxicity. Due to the clinical importance of assessing the vitamin D3 status in small animal patients, a quick, simple, and highly performing analytical method for its measurement is needed. In this study, we describe the development of a novel liquid chromatography–tandem mass spectrometry method for 25-hydroxyvitamin D3 quantification in canine serum. The approach was successfully validated following current European guidelines, proving excellent linearity (R2 always ≥0.996), accuracy (always within ±13%) and precision (always <10%). The application of the validated approach to samples collected from 40 healthy dogs made possible the definition of a reliable reference interval for 25-hydroxyvitamin D3, the main biomarker of vitamin D3. In addition, variations below 5% in the results obtained quantifying the same samples using a water-based calibration curve demonstrated that a surrogate matrix may be used without affecting data accuracy. Thanks to its simplicity, the proposed technique represents a useful tool for supporting clinical routine and investigating correlations between serum concentrations of this metabolite and multiple diseases. Additionally, it could enable the monitoring of supplementation in small animal patients in veterinary clinical practice.

## 1. Introduction

Small animals, such as dogs and cats, cannot synthesize enough vitamin D through their skin, probably due to the high activity of 7-dehydrocholesterol-Δ7-reductase [1,2]. Therefore, to meet their nutritional requirements, these animals are dependent on the dietary intake of vitamin D [3]. The two main dietary forms of vitamin D are cholecalciferol (vitamin D3) and ergocalciferol (vitamin D2), obtained from animal food sources and plant sources, respectively [4].

**Figure 1:**
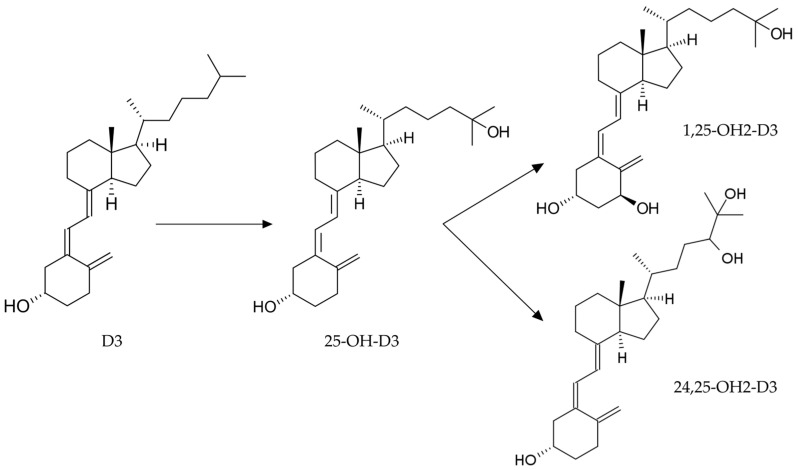
The metabolism of vitamin D3: at the liver level, it is converted to 25-OH-D3, which is then converted by the kidneys to 1,25-OH2-D3 and 24,25-OH2-D3.

After ingestion, vitamin D3 is quickly absorbed and converted into 25-hydroxyvitamin D3 (25-OH-D3, or calcidiol) by the hepatic 25-hydroxylase. In the bloodstream, calcidiol binds to vitamin D-binding protein (VDBP) and is transported to the kidneys. Its metabolic activity is achieved only through the conversion to 1,25-dihydroxy vitamin D3 (1,25-OH2-D3, or calcitriol) by 1α-hydroxylase [4,5] (see Figure 1). However, calcitriol is present at very low concentrations, has a short half-life, and is not stable during long-term storage [6]. The most reliable indicator of systemic vitamin D3 status is 25-OH-D3, which has a long half-life of approximately 2–3 weeks and is highly stable in stored serum samples [6,7,8].

Not only bone metabolism alterations, but also various disorders such as congestive heart failure, chronic enteropathy, chronic kidney diseases, certain types of cancer, and immune-mediated disorders have been associated with a low vitamin D3 status in dogs [4,9,10,11,12,13]. On the other hand, it is crucial to maintain balance and monitor the intake of this vitamin through the diet, as over-supplementation can lead to toxicity [14,15].

The clinical importance of monitoring vitamin status has led to an exponential increase in 25-OH-D3 testing. However, there is an ongoing debate regarding the appropriate reference intervals for interpreting 25-OH-D3 concentrations in canine serum, due to factors such as breed, age, and gender, which make it difficult to establish precise cut-off ranges. Additionally, a global interpretation of 25-OH-D3 is complicated by the variable accuracy of existing assays and the different population groups being analyzed. In daily practice, the most widely used methods are immunoassays, often automated, such as enzyme linked immunosorbent assay (ELISA), chemiluminescent immunoassay (CLIA), and radio immune assay (RIA) [16,17,18,19,20,21], or conventional high-performance liquid chromatography (HPLC) approaches coupled with ultraviolet (UV) or diode array (DAD) detectors [22,23]. Nevertheless, these approaches have limited analytical performance due to the lack of separation of vitamin D metabolites and are often subject to interferences.

Due to its sensitivity and analytical capacity, liquid chromatography–tandem mass spectrometry (LC–MS/MS) is increasingly becoming the technique of choice for diagnostics in human medicine. This high-throughput approach has recently started to spread in veterinary medicine, mainly for research purposes. Several studies quantified 25-OH-D3 in serum by LC–MS/MS, with the majority employing solid-phase extractions (SPE) [24,25], solid–liquid extractions (SLE) [3], and derivatizing agents [26,27], which are typically time-consuming and expensive.

Considering that 25-OH-D3 represents the best biomarker of vitamin D3 status in dogs, a simple and reliable approach would be beneficial for its measurement in serum samples also for routine purposes. The aim of this study was the development of a novel ultra-high liquid chromatography-tandem mass spectrometry (UHPLC–MS/MS) method for the quantification of 25-OH-D3 in canine serum. After validation, the technique was applied to the analysis of samples collected from 40 healthy donor dogs. This allowed us to define an appropriate reference interval based on this approach.

## 2. Materials and Methods

### 2.1. Serum Samples

The physiological reference interval for 25-OH-D3 was established by analyzing serum of healthy dogs (n = 40) previously collected at our Veterinary Hospital. The dogs were acknowledged blood donors, presented for an annual check-up, or dogs in which blood was sampled for pre-anesthetic evaluation before neutering. These dogs were eligible if they had no history or evidence of recent or chronic medical conditions and had not received any medication, except for routine vaccination and ectoparasite or endoparasite prophylaxis. The dogs were considered healthy due to no clinical or pathological evidence of disease according to an unremarkable history, physical examination and results of physical examination, complete blood count, biochemistry profile and urinalysis. Dogs were fed commercial maintenance diets and, according to their medical records, they did not receive any supplements containing vitamin D.

According to the sampling procedure, blood was collected from the jugular, saphenous or cephalic veins in serum separating tubes. Coagulated blood samples were centrifuged for 10 min at 3000× *g*; the serum was immediately transferred to plastic tubes, stored at −20 °C and thawed immediately before 25-OH-D3 analysis. Any plasma units with evidence of lipemia or hemolysis were previously excluded from the laboratory; therefore, only clear serum samples were available for 25-OH-D3 analysis.

The study was conducted in accordance with the Declaration of Helsinki, and the project was authorized by the Institutional Ethics Committee of Bologna’s University (protocol code 289564, and date of approval 9 October 2023).

The owners of all dogs signed an informed consent.

### 2.2. Chemicals and Reagents

Pure (>99%) analytical standard of 25-hydroxyvitamin D3 (25-OH-D3) at 100 µg/mL in ethanol and its isotopically labeled internal standard d6-25-hydroxyvitamin D3 (d6-25-OH-D3) at 50 µg/mL in ethanol were purchased from Sigma Aldrich (St. Louis, MO, USA). Ethyl acetate, hexane, methyl-tert-butyl ether (MTBE), zinc sulphate, as well as LC–MS grade acetonitrile, methanol, ultra-pure water, and formic acid were purchased from Sigma Aldrich (St. Louis, MO, USA).

### 2.3. Working Solutions 

Stock solutions were serially diluted in methanol to prepare the working solutions of 25-OH-D3 necessary for calibration and quality control (QC) samples. All solutions were protected from light (wrapped in aluminum foil). In particular, the working solutions had concentrations of 125, 250, 500, 1000, 1500, and 2000 ng/mL. All standard solutions were stored at −20 ± 2 °C in the dark.

### 2.4. Sample Preparation

After thawing at room temperature, 250 µL of serum sample was placed into a 2 mL Eppendorf microtube, adding 20 μL of internal standard working solution (d6-25-OH-D3 at 0.5 µg/mL in methanol) and 230 μL of methanol, and vortexing for 30 s. A liquid–liquid extraction (LLE) was carried out by adding 1.25 mL of ethyl acetate:hexane 75:25 (*v*/*v*) solution, vortexing for 1 min, and centrifuging at 21,000× *g* at 20 °C for 15 min. Then, 1.1 mL of the upper phase was transferred to a round bottom glass tube, and the LLE was repeated three times, adding 1.25 mL of ethyl acetate:hexane 75:25 (*v*/*v*) solution for each extraction. Then, a 4.4 mL aliquot of the upper phase was evaporated at 35 °C with nitrogen (N2) stream. The dry residue was reconstituted with 300 μL of mobile phase, which was a 15:85 (*v*/*v*) mixture of 0.1% formic acid water solution and methanol, agitated for 30 s on vortex mixer, and finally filtered with a 0.22 μm nylon syringe filter into a chromatography vial before the analysis.

### 2.5. Liquid Chromatography–Mass Spectrometry Conditions

Ultra-high performance liquid chromatography (UHPLC) was performed on a Waters Acquity UPLC^®^ system equipped with a binary pump, thermostatted autosampler, column oven, vacuum degasser, and condenser (Waters, Milford, MA, USA). Chromatographic separation was obtained with a Waters Acquity BEH C18 (50 × 2.1 mm, 1.7 µm) column protected by the corresponding VanGuard pre-column (Waters, Milford, MA, USA), and maintained at 35 °C. A satisfactory separation of 25-OH-D3 and d6-25-OH-D3 was obtained using a 2.5 min isocratic run combining water + 0.1% formic acid (solvent A) and methanol + 0.1% formic acid (solvent B), at a ratio of 15:85 (*v*:*v*) and a flow rate of 0.4 mL/min. Extracted samples were kept in the autosampler at 20 °C, and 7.5 µL from each vial was injected in the analytical system.

The UHPLC was interfaced to a Waters XEVO TQ-S Micro triple quadrupole mass spectrometer (Waters, Milford, MA, USA), operating in positive electrospray ionization (ESI+) and in multiple reaction monitoring (MRM) mode. Capillary voltage was set at 3.0 kV. Source and desolvation temperatures were 150 and 600 °C, respectively. Cone gas was set at 50 L/h, and desolvation gas at 900 L/h; argon was used as a collision gas. The analyte-dependent MS/MS parameters were optimized through combined infusion of LC mobile phase and standard solutions of each analyte at 0.5 μg/mL into the mass spectrometer. The most abundant ion transitions were 401.35 > 385.35 *m*/*z* (cone voltage 3V; collision energy 8 eV) and 401.35 > 365.24 *m*/*z* (3V; 9 eV) for 25-OH-D3 quantification and confirmation, respectively, and 407.4 > 389.28 *m*/*z* (3V; 7 eV) for d6-25-OH-D3. Data acquisition and analysis were performed with MassLynx 4.2 software (Waters, Milford, MA, USA).

### 2.6. Method Validation

Method validation was performed based on the European Medicines Agency ICH M10 guideline [28] during three different days of testing on serum and water. The following parameters were included: specificity, calibration range, accuracy, precision, extraction recovery (RE), matrix effect (ME), process efficiency (PE), carry-over, stability, and reinjection reproducibility.

#### 2.6.1. Specificity

The retentions times of 25-OH-D3 and its labelled internal standard d6-25-OH-D3 were defined by injecting pure standard at 100 ng/mL of each compound. Then, the specificity of the method was assessed first analyzing 10 water samples and mobile phases, to assess the absence of chromatographic signals at the retention times of 25-OH-D3 and d6-25-OH-D3. Then, mass chromatograms were inspected at the retention times of target analytes for potential interferences by other endogenous compounds in serum.

#### 2.6.2. Calibration Range

Calibration curves in pooled serum, including an unfortified sample (containing the endogenous concentration of 25-OH-D3) and six calibrators at 10, 20, 40, 80, 120, and 160 ng/mL, were prepared during each day of validation. Spiking was carried out by adding 20 μL of 25-OH-D3 of the corresponding working solutions. All samples were added with 20 μL of labelled internal standard d6-25-OH-D3 at a concentration of 0.5 µg/mL in methanol and extracted in the same manner as the unknown samples (see Section 2.3).

Peak area ratios between 25-OH-D3 and the corresponding internal standard were plotted against their concentration, and a linear least square regression model was applied. The endogenous concentration of the analyte was determined as the negative x-intercept of the calibration line. Background subtraction was then applied to correctly quantify unknown samples.

All calibration standards had to be within ±15% of the nominal value, and the resulting correlation coefficient (R2) was considered acceptable if ≥0.99. The lower limit of quantification (LLOQ) of the method was defined as the lowest endogenous concentration found that could be detected with a signal-to-noise (S/N) ratio ≥ 10 and acceptable accuracy and precision (<15%) after injection of four replicates.

In parallel, a neat solvent calibration curve and a calibration curve using water as surrogate matrix were prepared and analyzed during each session, to assess if measured levels of 25-OH-D3 in unknown samples were comparable to those obtained with matrix-matched calibration curves.

#### 2.6.3. Accuracy and Precision

To evaluate intra- and inter-day accuracy and precision of the method, QC samples at four different concentrations (10, 20, 80 and 120 ng/mL) were prepared in triplicate along with the calibration curve in serum during the 3 days of validation. Accuracy, expressed as relative difference between the measured value and expected concentration, was considered acceptable if within ±15%. Precision, defined as the coefficient of variation (CV%) among repeated individual measures, had to be <15% for each QC level.

Those parameters were also evaluated in water, preparing QCs samples at the same four concentrations.

#### 2.6.4. Recovery and Matrix Effect

For both analytes, individual post-column infusion was performed to evaluate ion suppression or enhancement. Serum samples (n = 6) with a low analyte concentration were injected while a solution of 25-OH-D3 or d6-25-OH-D3 at 160 ng/mL in mobile phase was infused into the mass spectrometer at 20 μL/min, to evaluate the stability of the produced signal.

The matrix effect was also calculated, as follows:

Matrix effect % = (Response_post-extracted sample_/Response_non-extracted neat sample_ − 1) × 100 (1)

Negative values imply ionization suppression, while positive values suggest an enhanced ionization.

Although d6-25-OH-D3 was used as internal standard, an evaluation of recovery, matrix effect, and process efficiency was performed following the approach reported by Matuszewski et al. 2006 [29]. Briefly, we compared peak areas obtained from three types of samples spiked at 160 ng/mL: one in the neat solvent (A, n = 6), one before the four-fold LLE extraction (B, n = 6), and one after the four-fold LLE extraction (C, n = 6). To evaluate RE, ME and PE, the following formulas were used: ME = B/A (%), RE = C/B (%), and PE = C/A (%) [29].

#### 2.6.5. Carry-Over

Following the injection of the highest calibrator (160 ng/mL), mobile phases were analyzed to assess the absence of carry-over.

#### 2.6.6. Stability

The stability of 25-OH-D3 in pooled serum obtained from six different subjects (healthy blood donors), was assessed after 1, 3 and 6 months at −20 °C. Extracted samples were reinjected after being left for 24 h in the autosampler at 20 °C, as well as after being frozen at −20 °C for 1 and 3 months.

We chose not to test shipping conditions, as this study was conducted in-house. As detailed in Section 2.1, serum samples were acquired from healthy blood donor dogs at the University Hospital’s blood bank and promptly stored at −20 °C until LC–MS/MS analysis, which was also performed within the same Department.

### 2.7. Statistical Analysis

Data obtained in healthy dogs were used to calculate the reference interval (RI) for 25-OH-D3 (ng/mL) using the Robust method, and 90% confidence intervals (CI) about their limits were bootstrapped as recommended for small sample sizes [30]. The values of 25-OH-D3 were tested for normality using the D’Agostino–Pearson test, and differences among sex and/or neutering status were tested with parametric statistics. Spearman’s correlation coefficient was used to assess correlations among 25-OH-D3, sex and/or neutering status. Statistical analyses were performed using available statistical software GraphPad Prism 10 and Microsoft Excel. Results were considered significant if *p* < 0.05.

## 3. Results

**Figure 2:**
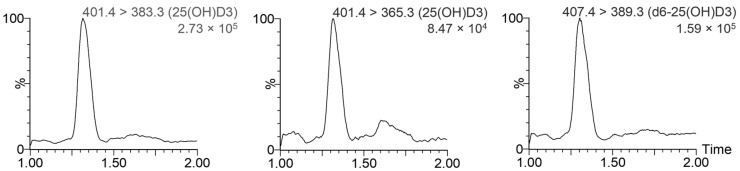
Chromatograms obtained from the MRM analysis of one of the serum samples collected from healthy dogs, including the ion transitions used for 25-OH-D3 quantification (**left**) and confirmation (**center**), and the ion transition monitored for the internal standard d6-25-OH-D3 (**right**).

The retention time for 25-OH-D3 and d6-25-OH-D3 was 1.31 min, as shown in Figure 2. The absence of interfering peaks at the retention times for target analytes after injection of mobile phase and water proved the specificity of the method. This was also confirmed by mass chromatograms without other endogenous compounds in serum in the same time window.

**Figure 3:**
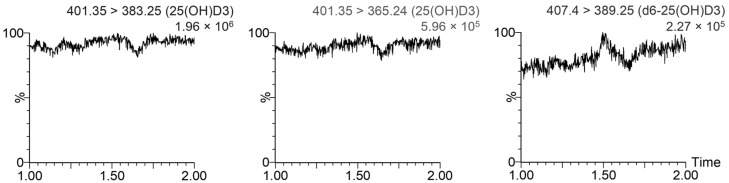
Evaluation of the matrix effect through observation of the signal produced by injecting a serum sample while infusing 160 ng/mL 25-OH-D3 (**left** and **center**) and d6-25-OH-D3 (**right**) standard solutions at 20 μL/min.

The lower limit of quantification (LLOQ) of the method for 25-OH-D3, defined as the lowest endogenous concentration found in the real samples that could be detected with a signal-to-noise (S/N) ratio ≥ 10 and acceptable accuracy and precision (<15%) after the injection of four replicates, was 10 ng/mL. The post-column infusion test did not show any matrix effect for 25-OH-D3 or for d6-25-OH-D3 (see Figure 3).

The data obtained from the additional tests confirmed the absence of a matrix effect (ME = 98.2%), together with the satisfactory extraction recovery (RE = 85.4%) and process efficiency (PE = 83.9%).

Calibration curves in pooled serum, water, and neat solvent were prepared on different days of testing. The coefficient of determination (R^2^) was always ≥0.99, and calibrators fell always within ±15% of the expected value, demonstrating the linearity of the method over the 10–160 ng/mL range of spiked concentrations. The three types of calibration curves were parallel with each other, as confirmed by the obtained slope values always differing by less than 10%.

At all QC levels in intra-day and inter-day conditions, accuracy and precision were always within ±13% and <10%, respectively (data shown in Table 1).

Measured concentrations in serum stored at −20 °C for 1, 3, and 6 months differed not more than 6% compared to those at day 0. Similarly, differences always within ±9% were obtained after reinjection of extracted samples left for 24 h in the autosampler at 20 °C and frozen at −20 °C for 1 and 3 months.

**Figure 4:**
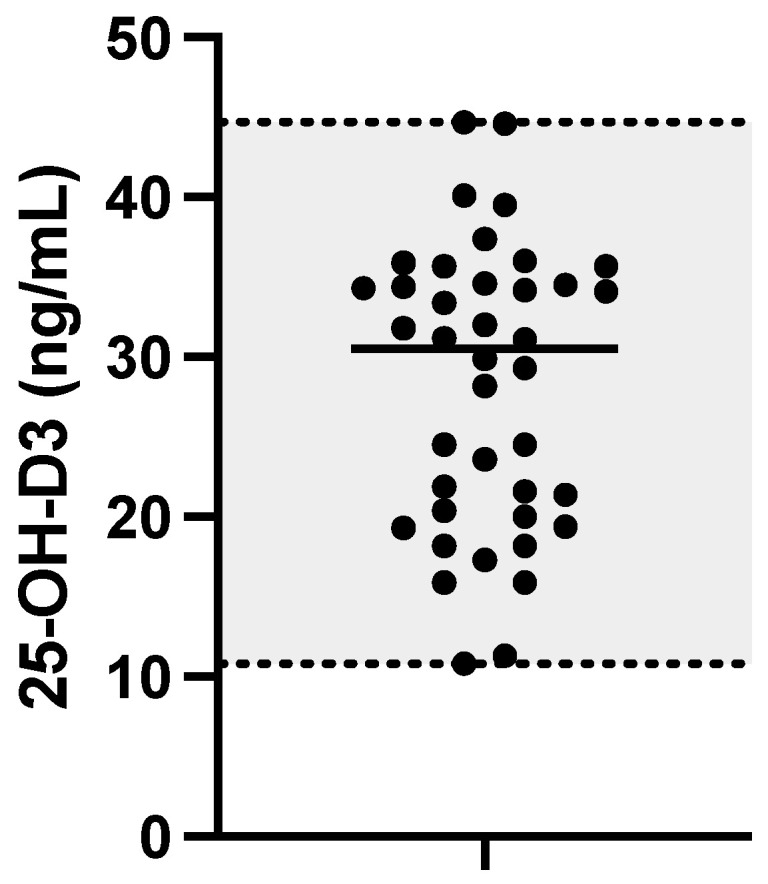
Dot plot showing results for 25-OH-D3 in healthy dogs. The black horizontal line represents the median. The grey-shaded area corresponds to the range minimum–maximum.

The concentration of 25-OH-D3 found in samples collected from 40 healthy dogs fell within the range of 10.8–44.7 ng/mL, with a median of 30.5 ng/mL. Significant differences in 25-OH-D3 according to sex or neutering status were not assessed (*p* > 0.05), nor correlations with variables such as age or body weight of dogs. For more detailed information, refer to Tables S1–S3 and Figure S1. The RI for 25-OH-D3 obtained in healthy dogs was 12.6 ng/mL for the lower limit (90% CI 9.2–17.8 ng/mL) and 44.7 ng/mL for the upper limit (90% CI 40.7–48.4 ng/mL) (see Figure 4).

## 4. Discussion

### 4.1. Method Development and Validation

Different mobile phase compositions, including water, acetonitrile, or methanol, with or without pH modifiers (e.g., formic acid, ammonium acetate, and ammonium formate), as well as gradient or isocratic eluent conditions were tested to optimize chromatography. The best resolution, peak shape, and intensity signal for 25-OH-D3 and d6-25-OH-D3 were obtained on a BEH C18 column under isocratic conditions of 15% water + 0.1% formic acid and 85% methanol + 0.1% formic acid flowing at 0.4 mL/min. In these conditions, the analytes eluted in 1.31 min. The chromatographic run of just 2.50 min allowed large batches of samples to be processed in a short time. Several organic solvents and extraction techniques, such as protein precipitation with 0.1 M zinc sulfate in water:methanol 30:70 (*v*/*v*), liquid–liquid extractions with various combinations of organic solvents (hexane:ethyl acetate 30:70 (*v*/*v*), hexane:ethyl acetate 90:10 (*v*/*v*), hexane:ethyl acetate 50:50 (*v*/*v*), 100% methyl-tert-butyl ether, and 100% hexane), were tested for sample treatment.

The best results in terms of recovery and background signal were obtained by applying a protein precipitation with methanol and a four-fold liquid–liquid extraction with 25:75 hexane:ethyl acetate (*v*/*v*) to 250 µL of serum. The optimized sample procedure allowed us to avoid more laborious and expensive extractions, such as solid-phase extraction [25] or solid–liquid extraction [31]. The chosen sample preparation procedure is quick and allows one to prepare 20 samples in just 1 h.

Thanks to the sensitivity of the proposed method, we were able to avoid the need for using derivatization agents [26,27]. These generally have high costs and make the sample treatment more complicated. Some of the published methods quantify 25-OH-D3 using stripped serum matrices or surrogate matrices, such as bovine serum albumin (BSA) [27,31,32]. In our study, during three separate days of testing, we prepared matrix-matched and unmatched calibration curves and QC samples, using pooled serum obtained from the animals enrolled in the study and water as a surrogate matrix, respectively. Both calibration curves (matrix-matched and unmatched) were then used to quantify the 40 samples from healthy blood donors (data shown in Table S4). Variations always below 5% demonstrated that water-based calibration curves may be used without affecting the accuracy of the data. In addition, the parallelism of the calibration curves prepared in pooled serum, water, and neat solvent suggested that any of these approaches can be used to reliably quantify unknown samples.

The high stability of 25-OH-D3 in matrix even after 6 months, as well as in extracted samples for 24 h in the autosampler at 20 °C and after being frozen at −20 °C for 1 and 3 months, makes it possible to collect and store samples for extended periods before analysis.

### 4.2. Dog Serum Analysis 

In this study, we established a reference interval for healthy dogs different in breed, sex, and weight. The range found here (10.8–44.7 ng/mL) closely resembled the reference intervals found in the literature. Parker et al. 2017 [4] reported a reference range of 24 to 86 ng/mL for 25-OH-D3 in a national veterinary endocrine laboratory. Other studies based on LC–MS/MS analysis indicated similar results, with mean concentrations in healthy dogs ranging from 29.6 to 57.0 ng/mL [32,33,34,35,36]. Additionally, quantifications of 25-OH-D3 in canine serum conducted using HPLC–UV [23,23] reported concentrations ranging from 17.2 to 30.76 ng/mL. However, direct comparison with other studies might be challenging, due to the difference in the analyzed populations and the analytical techniques employed. For example, various immunoassay methods such as CLIA, ELISA and RIA suggested a wider range of 25-OH-D3 concentrations in canine serum, spanning from 14.0 to 249.2 ng/mL [4,11,17,18,19,36,37,38].

This work has certain limitations that need to be addressed. Firstly, the small sample size, and the fact that the majority of dogs used were blood donors, with a body weight ≥ 20 kg by definition, could have led to results that reasonably might not be representative of the general client-owned dog population. Additionally, the dietary levels of 25-OH-D3, that is, the main vitamin D source in dogs, are unknown. In fact, those dogs were all fed different commercial dog foods for adult maintenance, which might contain anywhere from 500 to 3200 IU vitamin D/kg on a dry matter basis, according to FEDIAF Guidelines [39], depending on the level of vitamin D included by the manufacturers. In most cases, only the amount of added 25-OH-D3 is declared on the pet food label, while the amount from naturally contained ingredients is not accounted for on the label.

## 5. Conclusions

This study presents a novel and rapid UHPLC–MS/MS method for quantifying 25-OH-D3, the main biomarker of vitamin D, in canine serum. The proposed approach is both simple and highly efficient, offering a cost-effective and time-saving alternative to existing methods. After successful validation, it was firstly applied to 40 serum samples collected from both healthy dogs and blood donors, affording the chance to establish a reference interval in those subjects. This straightforward technique could serve in the near future as a valuable tool for exploring the relationship between serum concentrations of 25-OH-D3 and various diseases that are known to negatively affect the 25-OH-D3 status, including chronic conditions such as inflammatory enteropathy and neoplasia. Furthermore, it could be employed to monitor dietary and adjunctive supplementation of vitamin D in dogs that require it, particularly those affected by certain health conditions.

## Figures and Tables

**Table 1:** Intra-day and inter-day accuracy and precision of 25-OH-D3 in matrix-matched (pooled serum) and matrix-unmatched (water as a surrogate matrix) QC samples.

	Matrix-Matched (Pooled Serum)		Water (Surrogate Matrix)	
	Accuracy (%)	Precision (%)	Accuracy (%)	Precision (%)
	QC1 (10 ng/mL)		QC1 (10 ng/mL)	
Day 1 (n = 5)	0.0	1.0	−4.0	1.0
Day 2 (n = 5)	8.0	8.1	10.0	8.8
Day 3 (n = 5)	1.7	2.5	9.3	5.8
Inter-day (n = 15)	3.2	5.7	5.1	8.5
	QC2 (20 ng/mL)		QC2 (20 ng/mL)	
Day 1 (n = 5)	−1.3	4.1	3.2	5.2
Day 2 (n = 5)	−4.5	2.1	12.7	6.5
Day 3 (n = 5)	4.8	8.0	0.5	5.3
Inter-day (n = 15)	−0.3	6.3	5.1	7.5
	QC3 (80 ng/mL)		QC3 (80 ng/mL)	
Day 1 (n = 5)	0.9	1.9	−1.8	1.4
Day 2 (n = 5)	−4.8	1.6	4.1	9.3
Day 3 (n = 5)	−3.3	6.7	−2.5	4.2
Inter-day (n = 15)	−2.4	4.4	−0.1	6.1
	QC4 (120 ng/mL)		QC4 (120 ng/mL)	
Day 1 (n = 5)	−6.4	2.6	−5.8	1.5
Day 2 (n = 5)	−6.5	2.6	0.8	8.6
Day 3 (n = 5)	−0.9	1.5	−0.6	1.7
Inter-day (n = 15)	−4.6	3.5	−1.9	5.5

## Data Availability

The data presented in this study are available in the Supplementary Materials.

## Supplementary Materials

The following supporting information can be downloaded at: https://www.mdpi.com/article/10.3390/ani14010062/s1, Table S1: Study population (healthy dogs, n = 40) and serum levels of 25-OH-D3; Table S2: Hematological parameters in the study population (healthy dogs, n = 40); Table S3: Serum chemistry results in the study population (healthy dogs, n = 40); Table S4: Comparison between measurements with serum- and water-based calibration curves.; Figure S1: Correlation between 25-OH-D3 levels and patient’s sex (left), age (center), and body weight (right); Figure S2: Owner’s authorization form.
