# Supplementary material for: A Novel UHPLC–MS/MS Method for the Measurement of 25-Hydroxyvitamin D3 in Canine Serum and Its Application to Healthy Dogs
Source: Animals (Basel). 2023 Dec 23;14(1):62. doi: 10.3390/ani14010062 (PMC10778062; doi:10.3390/ani14010062)
Supplement: Supplementary file 1 [file animals-14-00062-s001.zip › animals-2708991-supplementary.pdf]

Supplementary Files

Table S1: Study population (healthy dogs, n = 40) and serum levels of 25-OH-D3.

Dog	Breed	Sex	Age (months)	Weight (kg)	25-OH-D3 (ng/mL)
1	Mixed breed	CM	49	40	18.2
2	Australian Shepherd	CM	71	26.5	31.1
3	Mixed breed	CM	49	41	17.3
4	Cane Corso	SF	64	56	31.2
5	Labrador retriever	SF	25	30	31.8
6	Labrador retriever	M	25	28.1	34.1
7	Boxer	SF	24	29.4	23.6
8	Mixed breed	M	28	31.3	18.2
9	Golden retriever	SF	50	27.7	10.8
10	Labrador retriever	M	76	29.5	32.0
11	Australian Shepherd	M	69	33	44.6
12	German Shepherd	CM	31	38.5	24.5
13	Mixed breed	SF	84	28.3	29.9
14	Boxer	SF	84	26.3	35.7
15	Golden retriever	SF	69	28.6	11.3
16	Leonberger	M	27	47.6	15.9
17	Golden retriever	F	63	30	33.4
18	Labrador retriever	SF	21	23	39.5
19	Golden retriever	F	44	25	21.4
20	Mixed breed	M	18	45	44.7
21	Labrador retriever	CM	14	36	19.3
22	Golden retriever	M	72	35.4	20.4
23	Flat-coated retriever	M	34	34.2	28.2
24	Mixed breed	SF	56	33.5	37.4
25	Labrador retriever	F	81	33.6	24.5
26	Maremma-abruzzese Sheepdog	CM	15	43.5	34.5
27	Mixed breed	CM	63	40.1	29.3
28	Mixed breed	SF	16	24	20.0
29	Saint Bernard	M	77	78	34.6
30	Golden retriever	F	56	31.1	21.9
31	Mixed breed	F	17	15.6	19.4
32	Mixed breed	M	12	7.2	34.2
33	Mixed breed	SF	12	23.5	34.3
34	Chihuahua	SF	15	3.2	15.9
35	Mixed breed	F	19	17.1	35.9
36	Mixed breed	F	12	3	36.0
37	Mixed breed	F	18	20.8	35.7
38	Parson Jack Russel terrier	F	14	6.8	34.4
39	Mixed breed	SF	17	24.4	21.6
40	Labrador retriever	M	37	32.8	40.1
**Mean**			**41**	**30.2**	**28.2**
**Median**			**33**	**29.8**	**30.5**
**Min**			**12**	**3.0**	**10.8**
**Max**			**84**	**78.0**	**44.7**
**Lower RI**					**12.6**
**90% CI**					**9.6-17.8**
**Upper RI**					**44.2**
**90 % CI**					**40.7-48.4**

CM: castrated male; F: female; M: male; SF: spayed female.

Table S2: Hematological parameters in the study population (healthy dogs, n = 40).

	Reference interval	Mean	Min	Max
RBC (x10^6/µl)	5.65-8.40	7.02	5.63	8.33
HGB (g/dl)	14-19	17	14.2	20.2
Hct (%)	39-58	49	39.9	58.5
MCV (fL)	63-77	70	62.6	76.7
MCHC (g/dl)	32-37	34.6	33	36.1
RDW (%)	11.6-14.6	13.2	11.6	14.9
WBC (x10^3/µl)	5-14	9.2	5.8	15.2
Neutrophils (/µl)	3,000-10,000	5,492	3,160	9,580
Lymphocytes (/µl)	900-4,000	2,709	1,150	4,980
Monocytes (/µl)	140-1,000	400	130	960
Eosinophyls (/µl)	10-1,010	566	10	1,190
Basophils (/µl)	0-80	17.6	10	80
PLT (x10^3/µl)	150-500	248	111	455
MPV (fL)	8.3-13.6	11.5	9.0	17
Reticulocytes (%)	0-1.5	0.82	0.24	2.15

Hb: haemoglobin concentration; HCT: haematocrit value; MCH: mean corpuscular haemoglobin; MCHC: mean cell haemoglobin concentration; MCV: mean corpuscular volume; MPV: mean platelet volume; PLT: platelet count; RBC: red blood cell count; RDW: red cell distribution width; WBC: white blood count.

Table S3: Serum chemistry results in the study population (healthy dogs, n = 40).

	Reference interval	Mean	Min	Max
AST (U/L)	15-52	34	22	66
ALT (U/L)	15-65	48	22	179
ALP (U/L)	12-180	39	8	87
Tot. cholesterol (mg/dL)*	123-345	256	149	345
Glucose (mg/dL)	65-115	98	50	119
Total protein (g/dL)	5-60-7.30	6.36	5.68	7.26
Albumin (g/dL)	2.75-3.85	3.24	2.70	3.54
Globulin (g/dL)	2.53-3.73	3.12	2.52	4.12
Alb/Glob	0.75-1.35	1.05	0.76	1.33
Urea (mg/dL)	17-48	35	21	59
Creatinine (mg/dL)	0.75-1.4	1.13	0.69	1.44
Phosphorus (mg/dL)	2.65-5.40	4.04	2.92	6.02
Calcium (mg/dL)	9.3-11	10.39	9.80	10.8
CaxP	< 60	42	31	60.8
Sodium (mEq/L)	143-151	148	144	154
Potassium (mEq/L)	3.8-5.0	4.5	4.0	5.3
Na/K	> 27	33	28	37
Chloride (mEq/L)	108-118	113.5	111	116
Magnesium (mg/dL)	1.7-2.35	2.0	1.66	2.27

Alb/Glob: albumin globulin ratio; ALP: alkaline phosphatase; ALT: alanine transaminase; AST: aspartate transaminase; CaxP: calcium phosphorus product; GGT: γ-Glutamyl transferase; Na/K: sodium potassium ratio. *For total cholesterol values from 20/40 dogs are available.

Table S4: Comparison between measurement with serum- and water-based calibration curves.

For each animal, the serum concentration calculated with a matrix-matched calibration curve and with a water-based calibration curve are reported, with the relative difference between the two measurements.

**Dog**	**25-OH-D3 (ng/mL)**	**25-OH-D3 (ng/mL)**	**Relative difference**
1	18.2	18.2	0%
2	31.1	30.5	-2%
3	17.3	16.4	-5%
4	31.2	30.0	-4%
5	31.8	30.9	-3%
6	34.1	33.8	-1%
7	23.6	23.0	-2%
8	18.2	17.2	-5%
9	10.8	10.4	-3%
10	32.0	30.5	-5%
11	44.6	44.5	0%
12	24.5	24.0	-2%
13	29.9	29.2	-2%
14	35.7	34.7	-3%
15	11.3	10.9	-3%
16	15.9	16.0	1%
17	33.4	32.4	-3%
18	39.5	38.1	-3%
19	21.4	21.4	0%
20	44.7	43.8	-2%
21	19.3	19.4	1%
22	20.4	19.8	-3%
23	28.2	28.7	2%
24	37.4	36.5	-2%
25	24.5	24.5	0%
26	34.5	34.3	-1%
27	29.3	28.9	-1%
28	20.0	19.8	-1%
29	34.6	33.3	-4%
30	21.9	21.5	-2%
31	19.4	18.8	-3%
32	34.2	34.0	-1%
33	34.3	32.8	-4%
34	15.9	15.1	-5%
35	35.9	34.2	-5%
36	36.0	35.9	0%
37	35.7	35.0	-2%
38	34.4	34.4	0%
39	21.6	21.6	0%
40	40.1	40.8	2%
**Mean**	**28.2**	**27.6**	**-2%**
**Median**	**30.5**	**29.6**	**-3%**
**Min**	**10.8**	**10.4**	
**Max**	**44.7**	**44.5**	

Figure S1: Distribution of 25-OH-D3 levels according to dogs' sex or neutering status (left). No significant differences were detected between groups (male n= 18 vs. female n= 22 and intact n= 19 vs. castrated n=21; Unpaired t-test, P > 0.05); Pearson's correlations between 25-OH-D3 levels and age in months (center), and body weight in kg (right).

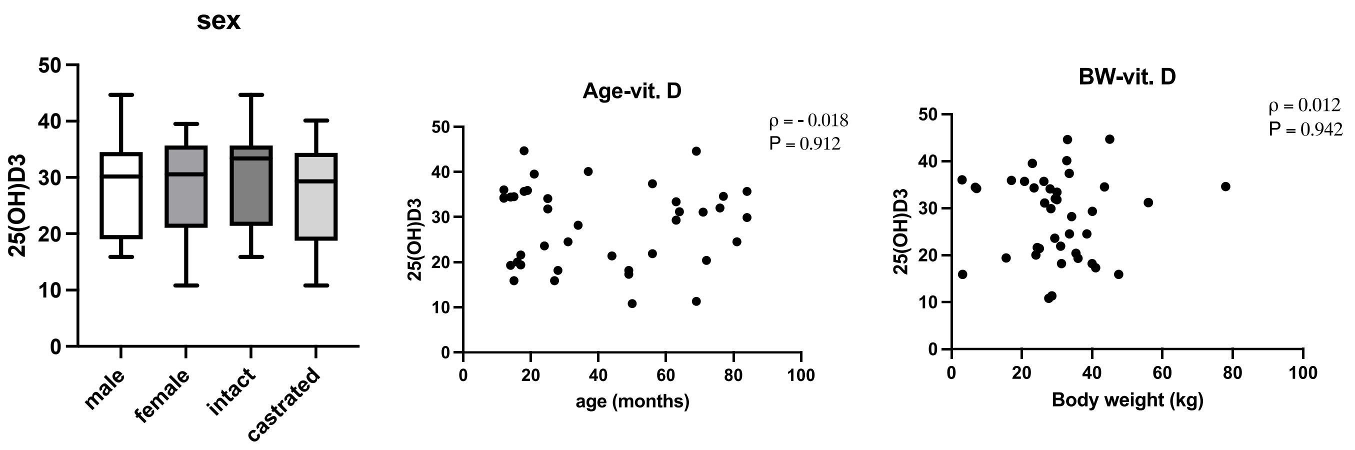

Figure S2: Owner's consent form

DIPARTIMENTO DI SCIENZE MEDICHE VETERINARIE
ALMA MATER STUDIORUM – UNIVERSITÀ DI BOLOGNA

Owner consent form

Owners name ________________________________, **Address**____________________________

____, **telephone number**_______________________,

dog's name________________________, **breed** ___________________________, **sex**_______,

date of birth ____/____/________, **microchip** ___________________________

I authorize the medical personnel to use excess stored serum samples from my dogs, beyond the amount required for the requested analyses, for other research purposes.

§ After carefully reading the above, I have signed in agreement.

____
(date and place)

____
(signature)
